# The Prolonged Application of Organic Fertilizers Increases the Quality and Yield of Tea Crops

**DOI:** 10.3390/plants14091317

**Published:** 2025-04-26

**Authors:** Cuiting Dai, Fen Xiang, Hongyan Liu, Lingyun Zhou, Wei Li

**Affiliations:** Hunan Tea Research Institute, Hunan Academy of Agricultural Sciences, Changsha 410125, China; hncysdai@hunaas.cn (C.D.); hncysxf@hunaas.cn (F.X.); hncyslhy@hunaas.cn (H.L.); hncyszly@hunaas.cn (L.Z.)

**Keywords:** organic fertilizer, soil quality, soil bacterial community, tea yield, tea quality

## Abstract

The substitution of chemical nitrogen (N) with organic fertilizers in tea plantations has been widely recognized as a strategy to maintain tea yield and improve soil quality, ensuring the sustainability of tea production systems. However, the effects of long-term organic-fertilizer substitution on tea yield and quality, soil properties, and bacterial communities have yet to be fully investigated, and the underlying mechanisms affecting tea yield and quality remain unclear. We conducted a six-year-long field experiment in a tea plantation to investigate the relationships among soil properties, bacterial communities, and the yield and quality of tea. Four treatments were compared: no fertilizer (NF), conventional fertilization (CF), 50% chemical N fertilizer substituted with a microbial organic fertilizer (MF), and 50% chemical N fertilizer substituted with a special organic fertilizer for tea (OF). The results showed that the substitution of organic fertilizers increased the spring tea yield by 6.4%~8.5% and the amino acid content of tea by up to 7.8%, while reducing tea polyphenol levels by 1.2–4.4% compared to CF. The soil quality improved significantly, with total phosphorus rising by 20.0% (MF) and 22.9% (OF), and soil organic matter increasing notably in the MF treatment group. The soil quality index (SQI) improved by 38.6% in the OF treatment group compared to the CF treatment group. Organic treatments reshaped bacterial communities, with the OF boosting *Acidobacteriota* (36.4%) and *Planctomycetota* (444.4%), and the MF enriching *Actinobacteria* and *Gemmatimonadetes*. Bacterial diversity (Shannon and Chao1 indices) correlated positively with the soil organic matter, total nitrogen, and pH. Changes in microbial communities were driven by pH, soil organic matter, and nitrogen levels. The partial least squares path model analysis confirmed that fertilization indirectly influenced tea yield (67% variance explained) and quality (79% variance explained) via soil properties and bacterial communities. These findings highlight the potential of organic-fertilizer substitution to promote sustainable tea production.

## 1. Introduction

Tea (*Camellia sinensis* L.) is one of the important economic crops in China. The planting area of tea plants in China has reached over 3.3 million hectares, with an annual output of over 3 million tons, both ranking first in the world [1]. As a leafy crop, the tea plant requires substantial nutrients to support growth and achieve high yields and quality. Fertilization is critical to meeting these demands [2]. To achieve high tea yields and significant economic returns, Chinese tea cultivators have persistently increased the use of chemical fertilizers. The application rates of nitrogen (N) fertilizers in Chinese tea plantations vary between 281 and 745 kg N ha^−1^ yr^−1^. Approximately 30% of tea plantations in China are burdened with the overuse of chemical N fertilizers, surpassing the recommended maximum application rate of 450 kg ha^−1^ yr^−1^ [3]. Long-term and excessive application of chemical fertilizers, especially N-based ones, negatively affect the soil quality and lead to soil degradation through soil acidification [4], soil structure deterioration [5], and biodiversity loss [6]. Thus, reducing synthetic N inputs has been recognized as an important topic for tea plantations.

Organic fertilizers are rich in nutrients and organic matter, which can enhance soil fertility and improve fertilizer utilization efficiency [7,8,9]. Therefore, organic fertilizers represent a viable alternative to chemical fertilizers. However, the exclusive use of organic fertilizers may lead to reduced crop yields due to their relatively low nutrient concentration and slow release rates [10,11]. To address this limitation, several studies have proposed strategies for the partial substitution of chemical fertilizers with organic fertilizers. Partial organic-fertilizer substitution has the potential to enhance crop yields and productivity by introducing exogenous carbon (C) inputs, thereby maintaining soil health [12]. Additionally, it can influence the loss of various reactive nitrogen (N) forms, such as N_2_O emissions, N runoff, and leaching, by modulating nitrogen transformation processes in the soil [13,14]. These positive effects are primarily attributed to the impact of organic amendments on the abundance, activity, and composition of soil microorganisms [15]. However, choosing appropriate organic amendments and understanding their effects on soil functions are important for advancing sustainable agricultural practices. Previous research has evaluated the influence of the partial substitution of chemical fertilizers with organic fertilizers on soil functions by focusing on specific indicators, such as the organic matter content, soil pH, and enzyme activities [16,17], which often neglects the integrated and synergistic interactions among various soil processes. Therefore, a comprehensive evaluation incorporating multiple soil indicators is necessary to accurately assess soil quality and agro-ecosystem functioning.

Soil quality is a synthesis of the capacity of soil to provide essential ecosystem functions and is critical for maintaining environmental quality and ensuring the sustainability of agricultural production systems [18,19]. The soil quality index (SQI) can be determined by integrating key soil physical, chemical, and biological properties. The influence of organic-fertilizer substitution on soil quality has been investigated across various agricultural systems. In tea plantations, maintaining soil fertility and health is a critical prerequisite for enhancing tea yield and quality. Existing studies in tea plantations have demonstrated strong correlations between organic-fertilizer substitution and soil quality, as well as between soil quality and tea yield [20]. However, most research on the effect of organic-fertilizer substitution in tea plantations has focused on individual physical or chemical indicators [21,22]. Overall, studies investigating the effects of long-term organic-fertilizer substitution on soil quality in tea plantations remain limited, particularly those involving comprehensive assessments of soil quality that consider multiple interacting factors.

Soil microorganisms play a crucial role in soil nutrient transformation and organic matter decomposition, and they are the core driving force for improving soil fertility, maintaining productivity and soil quality [23,24,25]. In tea production, positive associations have been observed between tea yields and some plant-growth-promoting microbes, suggesting the potential to enhance tea yield and quality by mediating soil microbial communities in tea plantations [26]. Organic fertilizers supply carbon, nitrogen, and energy to soil microbes [27], enhancing soil properties [28] and promoting microbial growth [29,30,31,32]. This increases microbial abundance and activity, improving community structure [33]. Long-term organic fertilization raises the content of soil organic matter and fertility, which are key drivers of microbial shifts [34]. By regulating soil pH and phosphorus levels, organic fertilizers increase beneficial microbes (e.g., *Proteobacteria*, *Bacteroidota*) linked to nutrient cycling, while inhibiting oligotrophic groups like *Verrucomicrobiota* [35]. These changes enhance microbial community stability and indirectly improve crop yields. Although the impact of organic fertilizers on soil fertility in tea plantations has been widely reported, differences in environmental conditions, tillage, soil quality, and climates in different regions lead to varying responses, especially in areas with acidic red soil in southern China where the literature on the effects of organic fertilizers on soil properties and bacterial communities is still limited.

Although numerous studies have investigated the response of yield to organic-fertilizer substitution in annual crops such as rice [36,37], maize [11], and wheat [38], research on perennial crops, particularly tea plants, remains limited. Some research suggests that an optimal organic-fertilizer-substitution ratio can effectively improve tea yield, increase the amino acid and catechin content [20], and mitigate soil acidification [39]. Despite these findings, the precise mechanisms through which organic-fertilizer substitution influences tea yield and quality remain poorly understood, and the relationship between tea yield and soil quality has not been thoroughly explored. Therefore, this study comprised a 6-year-long field experiment in a tea plantation with four different fertilization treatments: no fertilization (NF), conventional fertilization (CF), a 50% chemical N fertilizer substituted with a microbial organic fertilizer (MF), and a 50% chemical N fertilizer substituted with a special organic fertilizer for tea (OF), to investigate (1) the effects of different organic-fertilizer substitutions on tea yield and quality, (2) how the different organic-fertilizer substitutions change the soil properties and bacterial community structure, and (3) the influence of soil properties and bacterial communities on tea yield and quality and the corresponding mechanism underlying the improvement in tea yield. The study aimed to explore a suitable organic fertilization strategy for tea plantations and establish a theoretical foundation for the application of organic-fertilizer substitution, providing practical guidance for enhancing tea yield and soil quality in tea production.

## 2. Results

### 2.1. Effects of Organic-Fertilizer Substitution on Tea Yield and Quality

**Figure 1:**
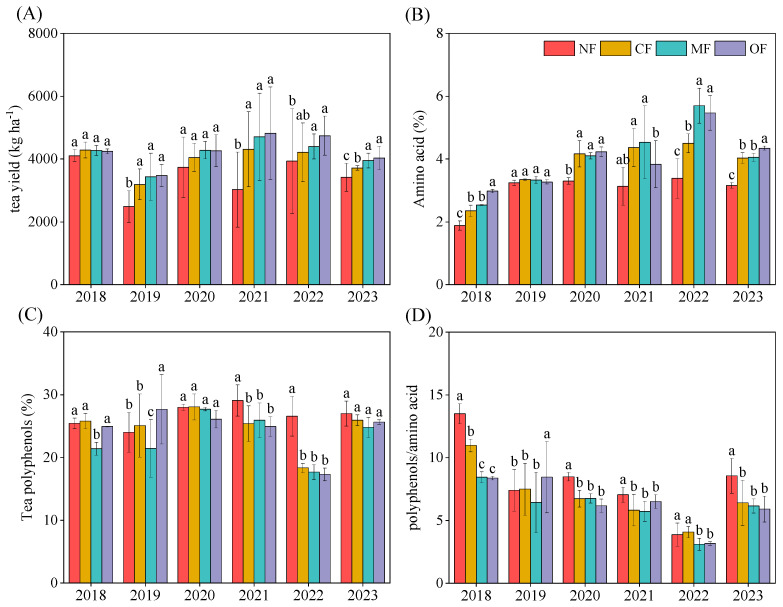
Tea yield (**A**), total amino acids (**B**), tea polyphenols (**C**), and ratio of tea polyphenols to amino acids (**D**) under different fertilization treatments. Different letters indicate significant treatment effects (*p* < 0.05). NF: no fertilization; CF: conventional fertilization; MF: 50% chemical N fertilizer substituted with microbial organic fertilizer; OF: 50% chemical N fertilizer substituted with special organic fertilizer for tea.

After 6 years of cultivation, the yield of spring tea without fertilization (NF) was 3416.47 kg ha^−1^ (Figure 1A). The highest yield of spring tea shoots was obtained with the OF treatment, followed by the MF treatment. Compared with CF, the MF and OF fertilization regimes significantly increased tea yield by 6.4% and 8.5%, respectively (*p* < 0.05). The spring tea yield in 2023 after the MF treatment was 3951.76 kg ha^−1^, but this value was not significantly different with that with the OF treatment (*p* >0.05). Organic-fertilizer substitution increased the amino acid content in spring tea with the cultivation duration. In 2023, organic-fertilizer substitution increased the amino acid content of spring tea by 0.6% to 7.8% compared with the CF treatment (Figure 1B). The highest amino acid content of spring tea was observed with the OF treatment, followed by the MF treatment. Organic-fertilizer substitution decreased the polyphenol content in spring tea during the experiment. By 2023, the polyphenol content in MF and OF treatment groups was 1.2% to 4.4% lower than with CF, but no significant differences were observed among the four treatments (*p* > 0.05; Figure 1C). Similarly, the polyphenol-to-amino-acid ratio in spring tea decreased with organic-fertilizer substitution. In 2023, the ratio for OF was 5.90, comparable to the CF and MF treatment groups, with no significant differences among the treatments (*p* > 0.05; Figure 1D).

### 2.2. Effects of Organic-Fertilizer Substitution on Soil Properties and the SQI

**Figure 2:**
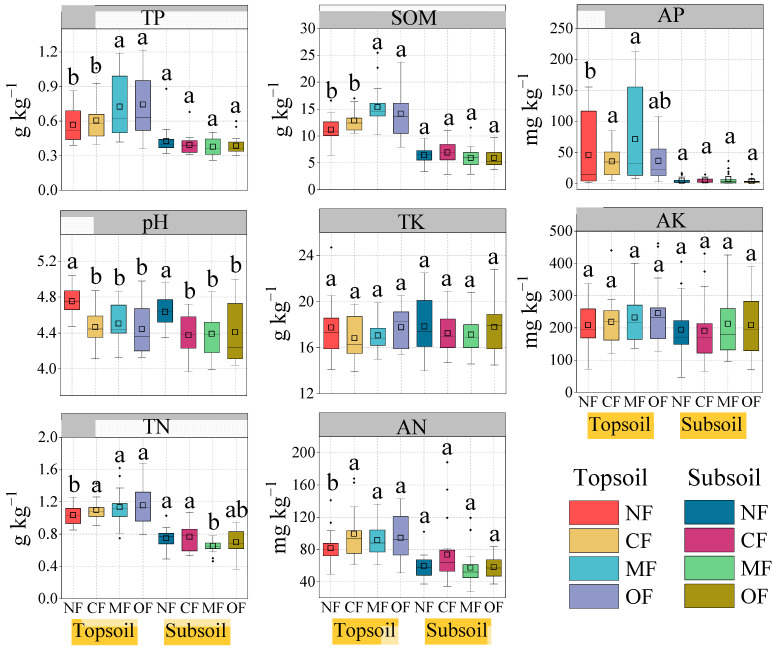
Topsoil (0–20 cm) and subsoil (20–40 cm) properties under different fertilization treatments during the experimental period. Soil organic matter (SOM), total nitrogen (TN), alkaline hydrolysis nitrogen (AN), total phosphorus (TP), available phosphorus (AP), total potassium (TK), available potassium (AK). Different letters indicate significant treatment effects (*p* < 0.05). NF: no fertilization; CF: conventional fertilization; MF: 50% chemical N fertilizer substituted with microbial organic fertilizer; OF: 50% chemical N fertilizer substituted with special organic fertilizer for tea.

**Figure 3:**
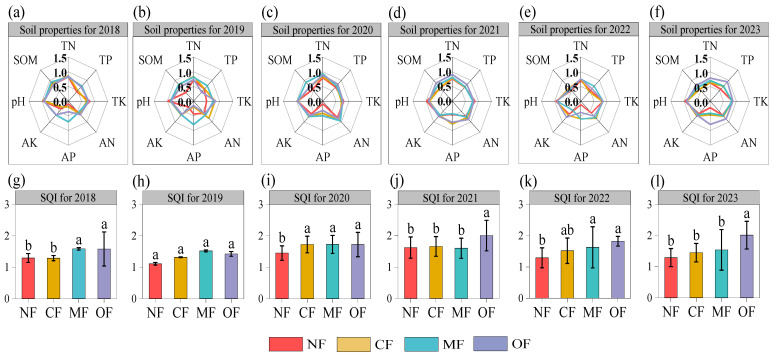
Radar graphs show the relative responses of soil properties to different fertilization treatments from 2018 to 2023 (**a**–**f**). NF: no fertilization; CF: conventional fertilization; MF: 50% chemical N fertilizer substituted with microbial organic fertilizer; OF: 50% chemical N fertilizer substituted with special organic fertilizer for tea. Soil organic matter (SOM), total nitrogen (TN), alkaline hydrolysis nitrogen (AN), total phosphorus (TP), available phosphorus (AP), total potassium (TK), available potassium (AK). Soil quality index (SQI) in response to fertilization treatments from 2018 to 2023 (**g**–**l**). Different letters indicate significant treatment effects (*p* < 0.05).

Organic-fertilizer-substitution treatments altered the contents of TP and SOM in the topsoil during the six-year-long fertilization management regime (Figure 2). Specifically, the TP contents of the topsoil in the MF and OF treatment groups were increased by 27.7% and 30.8%, respectively, compared to the NF treatment group. Compared with CF, the MF and OF treatments significantly increased the TP contents by 20.0% and 22.9%, respectively (*p* < 0.05). The SOM contents in the topsoil were significantly higher with the organic-fertilizer-substitution treatments (MF and OF) than conventional fertilization (CF), and the highest SOM value was observed in the MF-treated topsoil (*p* < 0.05). Compared with the NF treatment group, the topsoil and subsoil pH values were lower with the fertilization treatments, indicating that long-term fertilization acidified the tea-cultivated soil. Both the topsoil and subsoil pH values were not found to be significantly different between the MF and OF treatment groups. For the soil TK and AK, no significant differences were observed between the organic-fertilizer-substitution treatments (MF and OF) and CF in both the topsoil and subsoil. Compared with the NF treatment group, the fertilization treatments (CF, MF, and OF) significantly increased the topsoil TN by 6.0~11.6% and the AN by 12.1~21.9%. In 2023, the OF treatment significantly increased the topsoil SQI by 38.6% compared to CF. The MF treatment increased the topsoil SQI by 6.2%, but no significant difference was observed between the MF and CF treatment groups (Figure 3). In contrast, subsoil parameters were less affected, with only the pH showing a decrease under the fertilization treatments compared to the NF treatment group, and no significant differences in other subsoil properties between treatments were observed. Overall, organic-fertilizer-substitution treatments primarily improved the topsoil phosphorus, organic matter, and soil quality index values, with limited effects on subsoil properties.

### 2.3. Effects of Organic-Fertilizer Substitution on Soil Bacterial Community Diversity

**Figure 4:**
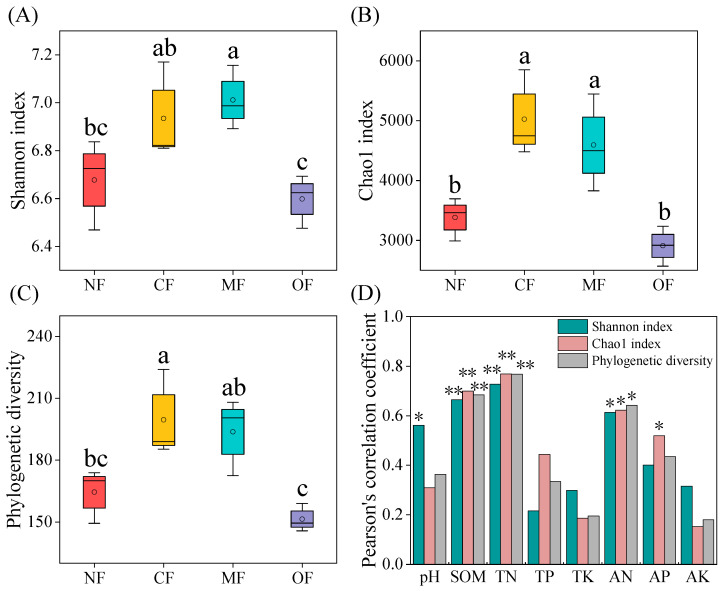
(**A**) Shannon index, (**B**) Chao1 index, and (**C**) phylogenetic diversity under different fertilization treatments. (**D**) Pearson’s correlation coefficients between alpha diversity and soil properties. Different letters indicate significant treatment effects (*p* < 0.05). NF: no fertilization; CF: conventional fertilization; MF: 50% chemical N fertilizer substituted with microbial organic fertilizer; OF: 50% chemical N fertilizer substituted with special organic fertilizer for tea. Soil organic matter (SOM), total nitrogen (TN), alkaline hydrolysis nitrogen (AN), total phosphorus (TP), available phosphorus (AP), total potassium (TK), available potassium (AK). * *p* < 0.05, ** *p* < 0.01.

Alpha diversities in soil bacterial communities were further explored by the Shannon, Chao1, and phylogenetic diversity indices. As is shown in Figure 4, the bacterial alpha diversity was significantly affected by fertilization treatments. Compared with CF, the OF treatment significantly decreased the Shannon index, Chao1, and phylogenetic diversity by 4.8%, 42.1%, and 24.1%, respectively. There were no significant differences showed in bacterial alpha diversity in the CF and MF treatment groups. Pearson correlation analysis revealed that the Shannon indices showed significant positive correlations with pH (*p* < 0.05), SOM (*p* < 0.01), TN (*p* < 0.01), and AN (*p* < 0.05); Chao1 indices showed significant positive correlations with SOM (*p* < 0.01), TN (*p* < 0.01), AN (*p* < 0.05), and AP (*p* < 0.05). Phylogenetic diversity showed positive correlations with SOM (*p* < 0.01), TN (*p* < 0.01), and AN (*p* < 0.05). In general, bacterial alpha diversity increased with increasing SOM, TN, and AN contents.

### 2.4. Shifts in Soil Bacterial Community Composition

**Figure 5:**
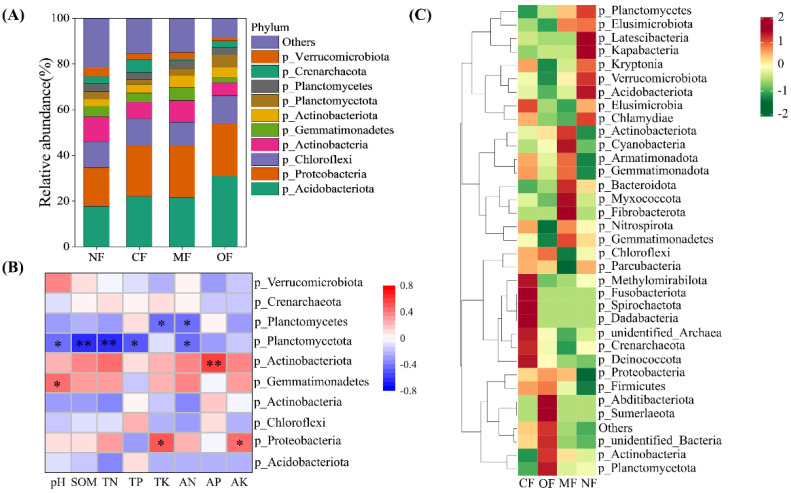
Relative abundances of soil bacterial communities at phylum level (**A**), Pearson’s correlation analysis between soil properties and top 10 bacterial phylum (**B**), and heatmap of the top 35 bacterial genera (**C**) under different fertilization treatments. NF: no fertilization; CF: conventional fertilization; MF: 50% chemical N fertilizer substituted with microbial organic fertilizer; OF: 50% chemical N fertilizer substituted with special organic fertilizer for tea. Soil organic matter (SOM), total nitrogen (TN), alkaline hydrolysis nitrogen (AN), total phosphorus (TP), available phosphorus (AP), total potassium (TK), available potassium (AK). * *p* < 0.05, ** *p* < 0.01.

The dominant phyla (> 1% average relative abundance) across all of the treatments were *Acidobacteriota* (21.5%), *Proteobacteria* (22.7%), *Chloroflexi* (9.8%), *Actinobacteria* (8.8%), *Gemmatimonadetes* (4.9%), *Actinobacteriota* (3.5%), *Planctomycetota* (3.7%), *Planctomycetes* (3.1%), and *Crenarchaeota* (3.1%), accounting for 81.1% of all of the bacterial sequences (Figure 5A). Compared with CF, the relative abundance of soil bacteria was changed in the soils of the MF and OF treatment groups. Specifically, *Acidobacteriota* was the dominant bacterial order in the soil of CF, but the OF treatment significantly increased its abundance by 36.4%. Compared with CF, the MF treatment significantly increased the abundance of *Actinobacteria* and *Gemmatimonadetes*, while the OF treatment significantly decreased the abundance of *Actinobacteria*. *Planctomycetota* abundance showed a significantly negative correlation with the soil pH, SOM, TN, TP, and AN (Figure 5B). The abundance of *Actinobacteriota* increased with the soil AN and *Proteobacteria* abundance increased with the soil AK and TK. The OF treatment significantly increased the abundance of *Planctomycetota* by 444.4%. Organic-fertilizer substitution enriched most soil genera (Figure 5C). Specifically, the OF treatment enriched the genera *p_Planctomycetota*, *p_Actinobacteria*, *p_Sumerlaeota*, and *p_Abditibacteriota*, whereas the MF treatment enriched the genera *p_Fibrobacterota*, *p_Myxococcota*, *p_Bacteroidota*, *p_Gemmatimonadota*, *p_Armatimonadota*, *p_Cyanobacteria*, and *p_Actinobacteriota*.

### 2.5. Relationships Among Soil Properties, Bacteria, and Tea Performance

**Figure 6:**
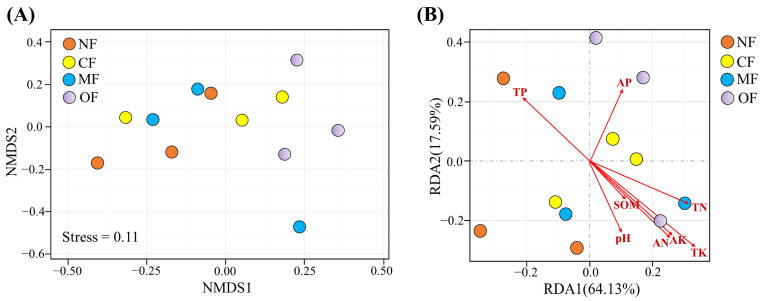
Non-metric multidimensional scaling (NMDS) based on the Bray–Curtis dissimilarities of bacterial communities under different fertilization treatments (**A**), Canonical redundancy analysis (RDA) of the environmental factors influencing bacterial community structure (**B**). NF: no fertilization; CF: conventional fertilization; MF: 50% chemical N fertilizer substituted with microbial organic fertilizer; OF: 50% chemical N fertilizer substituted with special organic fertilizer for tea. Soil organic matter (SOM), total nitrogen (TN), alkaline hydrolysis nitrogen (AN), total phosphorus (TP), available phosphorus (AP), total potassium (TK), available potassium (AK).

**Figure 7:**
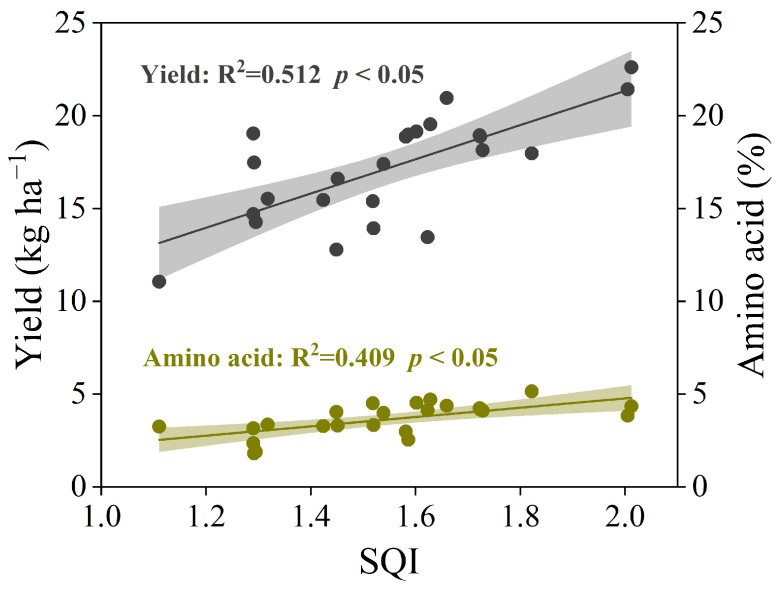
Relationship between yield, amino acid, and SQI by linear regression analysis.

Non-metric multidimensional scaling (NMDS) analysis indicated that organic-fertilizer-substitution treatment affected the structure of bacterial communities significantly (*p* < 0.05) (Figure 6A). The canonical redundancy analysis (RDA) further indicated that the structure of bacterial communities was mainly driven by the soil pH, SOM, TN, AN, and TK (Figure 6B). Pearson correlation analysis revealed that the tea yield and amino acid content were strongly correlated with the SQI (Figure 7).

**Figure 8:**
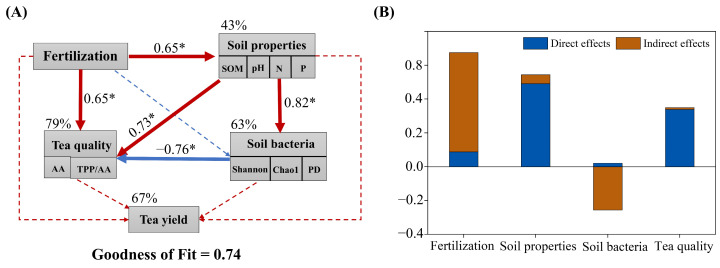
(**A**) Partial least squares path model (PLS-PM) analysis of relationships among fertilization treatments, soil properties, bacterial communities, tea yield, and quality. Observed or latent variables are illustrated in the box. Positive and negative effects are presented by red and blue arrows, respectively. Significant and nonsignificant effects are plotted by solid and dash lines, respectively. Path coefficients are presented on arrows. The goodness of fit was used to assess the model. * *p* < 0.05. Soil organic matter (SOM), amino acid (AA), tea polyphenols (TPP), ratio of tea polyphenols to amino acid (TPP/AA), phylogenetic diversity (PD). (**B**) Direct and indirect effects of the variables on tea yield based on the PLS-PM analysis.

A partial least squares path model (PLS-PM) analysis was constructed to determine the relationships among the fertilization treatments, soil properties, tea yield, soil bacterial communities, and tea quality (Figure 8A). The results showed that fertilization had a positive effect on the soil properties (0.65*) and tea quality (0.65*). The soil bacterial communities depended on the soil properties (0.82*). The soil properties (0.73*) determined the tea quality. Fertilization indirectly affected the tea yield by altering the soil properties and bacterial communities (Figure 8B). The 67% variation in the tea yield and 79% variation in the tea quality can be explained by the fertilization treatment and soil properties. The goodness of fit of the PLS-PM model was 0.74, which suggested that the PLS-PM model could be used to describe the relationships among fertilization treatments, soil properties, bacterial communities, tea yield, and quality.

## 3. Discussion

### 3.1. Organic-Fertilizer Substitutions Affect Soil Properties

The substitution of organic fertilizers has been confirmed to improve soil physical structure, increase soil nutrients, and optimize the soil microenvironment [40,41]. In this study, organic-fertilizer substitution significantly impacted the soil physicochemical properties, enhancing soil quality and promoting long-term agricultural sustainability by maintaining soil fertility. The results have shown that substituting chemical fertilizers with organic alternatives improved the soil organic matter (SOM), total nitrogen, and total phosphorus (Figure 2). Likewise, previous studies have demonstrated that organic-fertilizer substitution increased the SOM and soil fertility [42]. These changes in soil properties are attributed to several factors: (1) The SOM is an important indicator of soil fertility, and organic fertilizers enhance the transformation of organic matter, thereby improving SOM levels [43,44,45]. (2) The substitution of organic fertilizers promotes nutrient release from organic sources while reducing nutrient losses, leading to improved soil fertility [46,47]. (3) The combined application of chemical and organic fertilizers increases the C/N content, which stimulates soil microbial activity and enhances the micro-ecological environment [11]. Long-term application of chemical fertilizers often reduces the soil pH, contributing to soil acidification, particularly in tea cultivation systems. This study observed that soil pH decreased not only with conventional chemical fertilization but also with organic-fertilizer substitutions compared to the control group (no fertilization). This acidification may be influenced by multiple factors. Tea plant roots are known to excrete organic acids, such as oxalic and citric acids, which can lower the soil pH in the rhizosphere. Additionally, soil microbial activity may further influence pH, as certain microorganisms metabolize organic matter or nitrogenous compounds, releasing acidic byproducts like protons or organic acids. The extent of these effects can also vary depending on the type and substitution rate of organic fertilizers, as well as specific soil conditions and plant characteristics. Future research should disentangle the complex interplay of plant-, microbial-, and fertilizer-driven processes in shaping soil pH dynamics during tea cultivation.

In this study, the SQI, as an essential indicator of soil productivity, was evaluated to determine the effect of organic fertilization on soil properties. Previous studies have found that the SQI was improved significantly with increasing organic-fertilizer-substitution ratios (25~100%) but decreased under chemical fertilization in perennial tea plantations [20]. Our study demonstrated that 50% chemical N substitution with microbial organic fertilizer (MF), and 50% chemical N substitution with special organic fertilizer for tea (OF) increased the SQI (6.2~38.6%) after the six-year-long experiment (Figure 3). This result is consistent with the recent findings by the authors in [48], who found an increasing SQI in a tea plantation after two years of treatment with the partial substitution of chemical fertilizer with a biogas slurry and green manure. The enhanced SQI may due to the increase in SOM under organic-fertilizer substitutions. However, significant positive correlations have been observed between the SQI and tea yield and between tea amino acid content (Figure 7). These findings confirmed the benefits of organic-fertilizer substitution.

### 3.2. Organic-Fertilizer Substitutions Affects Yield and Quality of Tea

Organic-fertilizer substitution significantly enhances tea yield, with our study showing that organic-fertilizer substitution (OF and MF) increased yields by 6.4–8.5% after six years of cultivation compared to conventional fertilization (CF). This improvement is likely driven by the addition of exogenous organic matter in organic fertilizers, which increases the soil organic matter (SOM) content. SOM plays a critical role in supporting soil health and tea plant growth through multiple mechanisms. Firstly, higher SOM levels enhance the soil structure, and nutrient availability, creating optimal conditions for tea plant root development and nutrient uptake [49]. Secondly, SOM serves as a primary energy source for soil microorganisms, fostering a robust microbial community that facilitates nutrient cycling, particularly of carbon (C) and nitrogen (N). This microbial activity promotes the decomposition of organic matter, releasing metabolites that support tea plant physiological functions, including photosynthesis and C and N metabolism [50]. In our study, the increased SOM from organic fertilizers likely stimulated these processes, leading to the enhanced formation of metabolites critical for tea plant growth and yield. In addition, the results of the partial least squares path model analysis showed that fertilization was the most important factor affecting tea yield (Figure 8B). Tea polyphenols and amino acids are important indicators for evaluating the quality of tea [51]. The content of tea polyphenols determines the mellowness of tea and is an important health-promoting ingredient in tea. However, excessive content can also cause a bitter taste. The content of amino acids in tea leaves has a strong correlation with the tenderness of fresh leaves, and its content directly affects the fresh and refreshing taste of tea leaves [52,53]. The results of this study showed that the substitution of organic fertilizers could increase the content of amino acids in the leaves by 0.6% to 7.8% compared with CF (Figure 1B). A high amino acid content is conducive to the quality of green tea [54,55]. Organic-fertilizer treatments can reduce the content of tea polyphenols in the leaves by 1.2~4.4% compared with CF (Figure 1C). The polyphenol/amino acid ratio can not only be used as an indicator of the quality of tea, but is also one of the most important evaluation factors for the taste of green tea. The lower the polyphenol/amino acid ratio, the better the quality of green tea. Our study found that organic fertilization reduced the polyphenol/amino acid ratio and ultimately improved the quality of green tea.

### 3.3. Organic-Fertilizer Substitutions Affect Soil Bacteria

The alpha diversity index of soil bacteria is an important indicator for soil bacterial function and ecosystem stability [56]. Generally, the long-term application of chemical fertilizers leads to soil acidification and nutrient imbalance, reducing the soil bacterial diversity, while the application of organic fertilizers will stimulate the growth of soil bacteria and increase the soil bacterial diversity [57]. The authors in Ref. [58] conducted a 20-year-long field fertilization (organic and inorganic) experiment in China and found that in acidic soil areas with high precipitation and high soil fertility, the application of chemical fertilizers can lead to further acidification, which in turn has a negative impact on the soil bacterial diversity. Organic fertilizers can increase the bacterial abundance of most acidic soils [46]. In this study, compared with conventional fertilization, the MF treatment increased the alpha diversity index of bacterial communities, which is consistent with the findings of the authors in [59] in the Longjing Tea Garden. This may be due to microbial fertilizer improving the turnover of microorganisms and soil biological activity, forming a good environment conducive to bacterial growth and reproduction [60,61,62]. However, the OF treatment significantly decreased the alpha diversity index, which may be attributed to the decreased soil pH in the OF treatment group. Soil acidification reduces the number of ammonifying bacteria and nitrogen-fixing bacteria, affecting the growth and activity of these microorganisms [63,64]. In this study, the soil pH, total nitrogen, organic matter and available nitrogen are important factors correlating with the soil bacterial diversity (Figure 4D). Changes in the soil environment caused by different fertilization treatments significantly affect the composition of the bacterial communities [65]. The results of this study showed that the dominant bacterial phyla with different fertilization treatments were *Acidobacteriota*, *Proteobacteria*, *Chloroflexi*, *Actinobacteria*, and *Gemmatimonadetes*, which were consistent with previous studies [26,66]. *Proteobacteria* prefer a eutrophic environment and their abundance in our study was higher with the MF treatment than the CF treatment. The OF treatment significantly increased the abundance of *Acidobacteriota*, which may due to *Acidobacteriota* being acidophilic bacteria, and the soil pH in the OF treatment group decreased. Although the dominant soil bacterial phylum with different fertilization treatments is basically similar, there are significant differences in the overall composition of the soil bacterial community structure in each fertilization treatment according to the NMDS analysis. The RDA indicated that bacterial community structure was mainly driven by the soil pH, SOM, TN, AN and TK. Our partial least squares path modeling results found that the application of organic-fertilizer-substitution regimes may indirectly affect soil bacteria communities by changing the soil properties (Figure 8). Thus, this study confirmed the importance of soil bacteria in facilitating soil health and maintaining soil fertility [67].

## 4. Materials and Methods

### 4.1. Site Description and Experiment Design

The experimental tea plantation was located in Gaoqiao County of Changsha city, Hunan province, China (113°20′40″ E, 28°28′39″ N), which has a monsoon climate with an average annual precipitation of 1422 mm and an average annual temperature of 16.8 °C. The soil type is categorized as acidic red soil (Ultisols) formed from quaternary red clay, with a texture classified as loamy clay. The main properties of the initial soil are described in Table 1.

The field experiment was initiated in 2017 and included four fertilization treatments: (1) NF: no fertilization; (2) CF: conventional fertilization; (3) MF: 50% chemical N fertilizer substituted with microbial organic fertilizer; and (4) OF: 50% chemical N fertilizer substituted with special organic fertilizer for tea. The organic-fertilizer-substitution rate was set at 50% due to a previous field experiment revealing that the optimal soil nutrient availability, tea quality, and yield were achieved at a substitution ratio of 20–50% [68]. The chemical fertilizers were supplied as urea and formula fertilizer (N: P_2_O_5_: K_2_O = 24:11:10). The organic fertilizers applied were microbial organic fertilizer (N: P_2_O_5_: K_2_O = 5.5:5.8:5.8, derived from composted manure inoculated with *Bacillus subtilis* and *Trichoderma* spp.), and special organic fertilizer for tea (N: P_2_O_5_: K_2_O = 11:6:7.6, composed of composted tea residues, soybean meal, and bone meal). The amount of fertilizer applied is displayed in Table 2. All fertilizers were applied in tea furrows at a soil depth of 20 cm once a year in November. The area of each treatment was 150 m^2^. All treatments were conducted with three replicates using a completely randomized block design. Sprinkling irrigation was used to standardize soil moisture during periods of low precipitation. The tea plants were planted in 2009, and they were of the cultivar “Zhuyeqi” (*Camellia sinensis* L.), which was mainly used to produce green tea.

### 4.2. Soil Sampling and Analysis

Soil samples with a depth of 0–20 cm (topsoil) and 20–40 cm (subsoil) were collected in late October each year before fertilization. Five sampling points about 10 cm away from the fertilization ditch were selected randomly for the collection of soil samples in each treatment plot. The collected soil samples were sieved (<2 mm) and stored at −4 °C for soil physiochemical property analysis. One part of the topsoil samples was stored at −80 °C for soil DNA extraction.

The soil pH was measured with an ORION STAR A211 pH meter (Thermo Scientific Lab, USA) using a 1:2.5 (wt/vol) ratio of soil in double-distilled H_2_O. Soil organic matter (SOM) and total nitrogen (TN) were measured by using the elemental analyzer (Vario Max, Elementar, Germany). Total potassium (TK) and total phosphorus (TP) were measured after being treated by a H_2_SO_4_–H_2_O_2_ mixture. TK was measured using flame atomic spectrophotometry. TP was determined using an autoAnalyser3 (Bran + Luebbe, Hamburg, Langenselbold, Germany). Alkali-hydrolyzed nitrogen (AN), available potassium (AK), and available phosphorus (AP) were analyzed by the diffusion method, the ammonium acetate extraction flame photometry method, and the Olsen method, respectively.

### 4.3. Soil Quality Evaluation

All soil property indices were converted into dimensionless scores ranging from 0 to 1 to determine the soil quality index (SQI) for four fertilization treatments, as described below:

$${SL}_{i}=\frac{X}{X_{max}}$$

where *SL_i_* represents the dimensionless score of indicator *i* between 0 and 1; *X* and *X_max_* are the measured values and maximum value of indicator *i*, respectively. The SQI score was evaluated using an SQI area method based on the area of the radar diagram derived from the transformed soil indicators [49].

$${SQI}_{area}=0.5\times \sum_{i}^{n}{SL}_{i}^{2}\times \sin\frac{2\pi }{n}$$

where *SQI_area_* represents the SQI score and *n* represents the number of indicators used for calculating *SQI_area_*.

### 4.4. Yield and Quality of Tea

Tea shoots with one bud and one leaf were collected by hand in the spring, and their fresh weights were used to calculate the tea yield. The amino acid (AA) and total polyphenol (TPP) contents were used to represent the tea quality. Tea shoots were oven-dried and then ground before measuring their chemical properties. AAs and TPPs were extracted from 60 mg of the ground tea shoot powder and then soaked for 5 min in 3 mL of boiling ddH_2_O in a water bath, followed by vortex mixing for 2.5 min. The AA concentration was measured using glutamic acid as a reference standard and the TPP concentration was spectrophotometrically measured by the Folin–Ciocalteu colorimetric method using a *Genesys 10s* Spectrophotometer (Thermo Fisher Scientific, Waltham, MA, USA), with gallic acid used as the reference standard [60].

### 4.5. Soil DNA Extraction and High-Throughput Sequencing

Total soil DNA was extracted from 0.25 g of fresh soil using a soil DNA kit (Omega Bio-Tek, Inc., Norcross, GA, USA). All experimental operations were conducted according to the product’s protocol using a UV-sterilized ultraclean bench to avoid interference from external bacteria and impurities during the extraction. The quality and quantity of extraction were examined by the resulting DNA using a nano-spectrophotometer (ND2000, Thermo Scientific, Waltham, MA, USA) and gel electrophoresis (1% agarose gel, 0.5× tris-borate EDTA buffer), respectively.

The bacterial 16s rRNA (V4–V5 hypervariable region) was amplified using the primers 525F (5′-GTGCCAGCMGCCGCGG-3′) and 907R (5′-CCGTCAATTCMTTTRAGTTT-3′). The polymerase chain reaction (PCR) mixture (25 μL) contained 1 μL of the purified template DNA, 2.5 μL of 10 × PCR Mg^2+^ free buffer, 2.5 μL of 2.5 mM Mg^2+^, 2.0 μL of 25 mM dNTPs, 0.5 μL (1.25 U) of Taq polymerase, 0.5 μL (10 μM) of each primer, and sterilized ultrapure water up to 25 μL. The bacterial V4–V5-region amplification was carried out as follows: an initial denaturation at 94 °C for 5 min, 15 cycles of 94 °C for 60 s, 54 °C for 30 s, 72 °C for 90 s, and a final extension step at 72 °C for 10 min. The PCR was performed by a Thermal Cycler (ABI 2720, Thermo Fisher Scientific, USA). The PCR products were then purified using a DNA Gel Extraction Kit (QIAquick, Qiagen, Germantown, MD, USA).

The purified amplicons were sequenced using the Illumina MiSeq platform. Raw sequence data were initially trimmed to remove primers and barcodes, and then filtered using the software package “QIIME 1.9.1” and the UPARSE pipeline as implemented in USEARCH v8.0.1623. Chimeras were checked and removed. The high-quality sequences with ≥97% similarity were then clustered into operational taxonomic units (OTUs). The representative sequence chosen was based on the most abundant sequence for each OTU. OTU representative sequences were classified against the ribosomal database project (RDP) 16s rRNA database for bacteria with an 80% confidence threshold. In total, 17,184,727 (153,378 ± 6 9882 per sample) high-quality sequences were obtained after quality control. To accurately assess the diversity of the bacterial communities, the number of sequences were normalized to 55,221 sequences per sample for further analysis. The α-diversity (including Chao1 and Shannon indices) of soil bacteria was calculated by the Mothur software (version 1.48.0).

### 4.6. Statistical Analysis

The Kolmogorov–Smirnov normality test was used to assess the normality of the data for each set of physicochemical factors, yield and quality of tea, and microbial community structure. Data that followed a normal distribution were analyzed by a one-way analysis of variance (ANOVA) with a Least Significant Difference test (*p* < 0.05) using IBM SPSS Statistics 27 software (IBM Corporation, Armonk, NY, USA). A Pearson’s correlation analysis was applied to evaluate the relationships between soil properties and soil bacterial community compositions. Linear regression analysis was used to reveal the relationships between the soil quality index and yield and the amino acid content of tea. Canonical redundancy analysis (RDA) was applied by using R package “Vegan” to investigate the effects of environmental factors on the bacterial community structure. The partial least squares path model (PLS-PM) was applied by using the “plspm” package in R to analyze the relationships among organic fertilization, soil properties, bacterial communities, tea yield, and quality. Soil properties were indicated by soil organic matter, total nitrogen, total phosphorus, and pH. A total of 1000 bootstraps were used to calculate path coefficients and coefficients of determination (R^2^). The overall prediction performance of the model was assessed using the goodness-of-fit index. The statistical analyses were performed on the R platform (version 4.4.0).

## 5. Conclusions

Our study investigated the effects of organic-fertilizer substitution on tea yield and quality, soil properties, and soil bacteria over a six-year-long experiment. Our findings reveal that organic-fertilizer applications enhance tea production by improving soil fertility and microbial diversity, which in turn support higher yields and better tea quality. Specifically, organic fertilizers lower soil acidity and increase organic matter and nutrient availability, creating a more favorable environment for tea plant growth. These soil changes foster diverse bacterial communities, including taxa that likely contribute to nutrient cycling and soil health, indirectly influencing tea performance. The scientific contribution of this work lies in demonstrating that organic-fertilizer substitution not only sustains but enhances tea plantation productivity, offering a practical strategy for sustainable agriculture. However, this study is limited by its focus on a single tea plantation, which may not fully represent diverse agroecological conditions. Future studies could explore optimal organic-fertilizer formulations, long-term impacts on different tea varieties and regions, and the role of microbial functional genes in nutrient transformations.

## Figures and Tables

**Table 1:** Main properties of initial soil in this study (mean ± standard deviation).

Property	0–20 cm	20–40 cm
pH	4.94 ± 0.37	4.91 ± 0.41
TN (g kg^−1^)	0.78 ± 0.02	0.61 ± 0.05
TP (g kg^−1^)	0.37 ± 0.15	0.34 ± 0.02
TK (g kg^−1^)	1.67 ± 0.17	1.60 ± 0.34
SOM (g kg^−1^)	10.30 ± 1.59	8.24 ± 1.28
AN (mg kg^−1^)	58.60 ± 7.74	90.4 ± 12.33
AP (mg kg^−1^)	10.40 ± 2.15	5.72 ± 0.39
AK (mg kg^−1^)	382.00 ± 32.48	300.00 ± 25.75

Note: TN: total nitrogen, TP: total phosphorous, TK: total potassium, SOM: soil organic matter, AN: available nitrogen, AP: available phosphorous, AK: available potassium.

**Table 2:** Description for each fertilization management regime.

Treatment	Description	Chemical N Amount (Kg Ha^−1^)	Organic N Amount (Kg ha^−1^)	OSR (%)
NF	No fertilization	0	0	—
CF	Conventional fertilization	777	0	—
MF	Microbial organic fertilizer + formula fertilizer + urea	387	165	50
OF	Special organic fertilizer + formula fertilizer +urea	387	165	50

Note: OSR represents organic-fertilizer-substitution rate for chemical N fertilizer.

## Data Availability

Data are contained within the article.
